# Phylogenomics of a putatively convergent novelty: did hypertrophied lips evolve once or repeatedly in Lake Malawi cichlid fishes?

**DOI:** 10.1186/s12862-018-1296-9

**Published:** 2018-11-29

**Authors:** C. Darrin Hulsey, Jimmy Zheng, Roi Holzman, Michael E. Alfaro, Melisa Olave, Axel Meyer

**Affiliations:** 1 Department of Biology, University of Konstanz, Konstanz, Germany; 2 Department of Ecology & Evolutionary Biology, University of California, Los Angeles, CA USA; 3 Department of Zoology, Tel Aviv University and the Inter-University Institute for Marine Sciences in Eilat, 88103 Eilat, Israel

**Keywords:** Adaptive radiation, East African Rift Lakes, Fatlips, Phylogenomics

## Abstract

**Background:**

Phylogenies provide critical information about convergence during adaptive radiation. To test whether there have been multiple origins of a distinctive trophic phenotype in one of the most rapidly radiating groups known, we used ultra-conserved elements (UCEs) to examine the evolutionary affinities of Lake Malawi cichlids lineages exhibiting greatly hypertrophied lips.

**Results:**

The hypertrophied lip cichlids *Cheilochromis euchilus, Eclectochromis ornatus, Placidochromis “*Mbenji fatlip*”,* and *Placidochromis milomo* are all nested within the non-mbuna clade of Malawi cichlids based on both concatenated sequence and single nucleotide polymorphism (SNP) inferred phylogenies. *Lichnochromis acuticeps* that exhibits slightly hypertrophied lips also appears to have evolutionary affinities to this group. However, *Chilotilapia rhoadesii* that lacks hypertrophied lips was recovered as nested within the species *Cheilochromis euchilus.* Species tree reconstructions and analyses of introgression provided largely ambiguous patterns of Malawi cichlid evolution.

**Conclusions:**

Contrary to mitochondrial DNA phylogenies, bifurcating trees based on our 1024 UCE loci supported close affinities of Lake Malawi lineages with hypertrophied lips. However, incomplete lineage sorting in Malawi tends to render these inferences more tenuous. Phylogenomic analyses will continue to provide powerful inferences about whether phenotypic novelties arose once or multiple times during adaptive radiation.

**Electronic supplementary material:**

The online version of this article (10.1186/s12862-018-1296-9) contains supplementary material, which is available to authorized users.

## Background

Phylogenies are critical to testing convergence. Evolutionary trees can provide the framework for determining whether similar phenotypes have multiple origins or whether these traits have arisen only a single time [1–6]. Molecular phylogenies of East African cichlids provided some of the first examples of using DNA sequence data to establish the repeated evolution of similar phenotypes in radiations inhabiting different lakes [7–10]. Yet, clarifying whether cichlid lineages have evolved traits convergently within particular cichlid radiations that inhabit the same lake has remained problematic [11–16]. Additionally, the short periods (< 2 mya) over which cichlid groups like those inhabiting Lake Victoria and Lake Malawi have diversified have made reconstructing the phylogenies of these adaptively radiating groups exceptionally difficult [17–23]. However, phylogenomic analyses could allow us to determine whether traits such as the elongate bodies of piscivores, distinctive color patterns of fish inhabiting rocky reefs, or even bizarre phenotypes like greatly hypertrophied lips have arisen multiple times in radiations like the Lake Malawi cichlids.

**Fig. 1:**
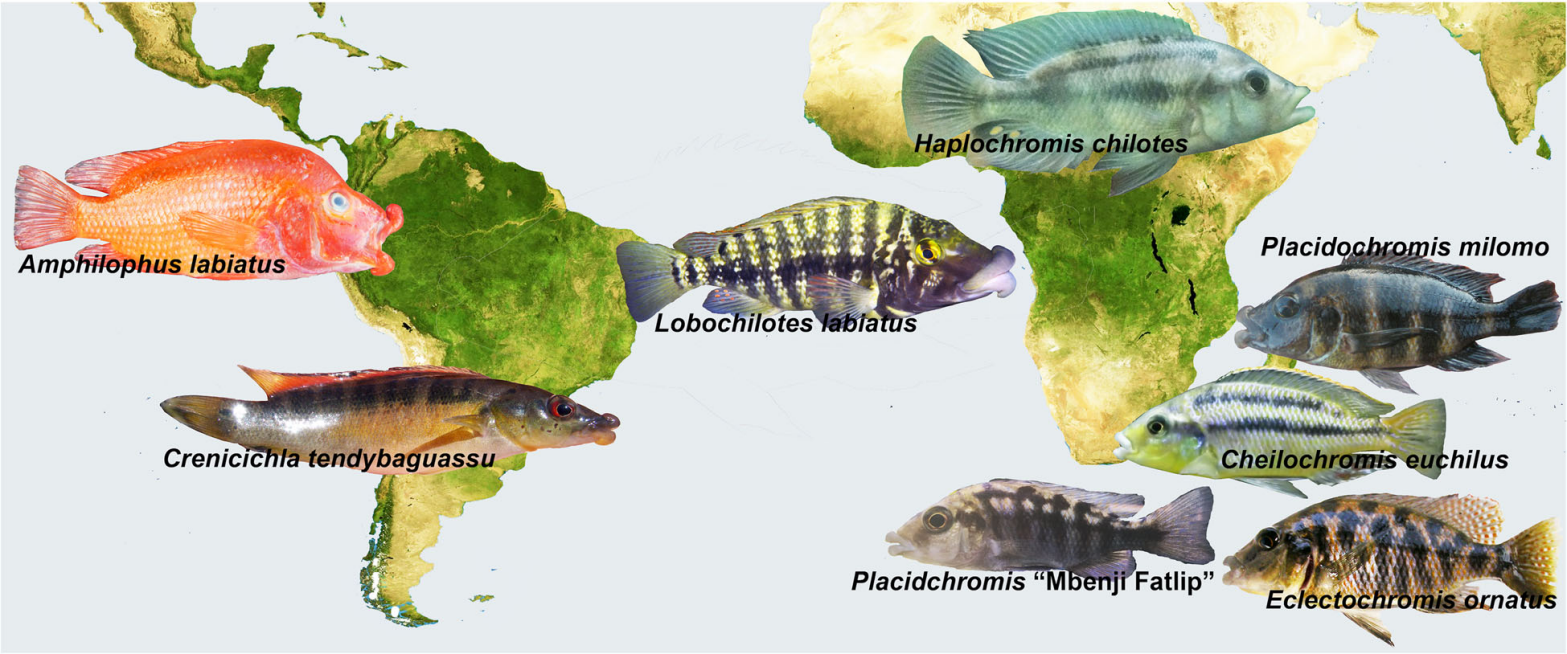
Convergent evolution of hypertrophied lip phenotypes in cichlids fishes. This phenotype has arisen independently in a number of cichlid lineages inhabiting regions ranging from the Nicaraguan Rift Lakes (*Amphilophus labiatus*), South America (*Crenicichla tendybaguassu*), Lake Tanganyika (*Lobochilotes labiatus*), Lake Victoria (*Haplochromis chilotes*), and Lake Malawi (*Cheilochromis euchilus, Eclectochromis ornatus, Placidochromis “*Mbenji fatlip*”,* and *Placidochromis milomo*). The lineages containing hypertrophied lips from these disparate geographic settings outside of Lake Malawi are well established as phylogenetically independent [79]. However, it is unclear if the hypertrophied lip phenotype has arisen convergently multiple times or alternatively only once within Lake Malawi

Hypertrophied, or greatly enlarged, lips have arisen several times independently across fish diversity. Lineages possessing hypertrophied lips are present in the sailfin silverside fishes from the Malili lakes of Sulawesi [24] as well as in the barb species inhabiting Lake Tana of Ethiopia [25, 26]. But the best-known group that exhibits hypertrophied lips are the cichlid fishes (Fig. 1). Cichlids exhibiting greatly hypertrophied lips have independently arisen in the Nicaraguan crater lakes, large South American rivers, Lake Tanganyika, Lake Victoria, and Lake Malawi [10, 27–33]. This phenotype is often associated with feeding from rocky surfaces, and is likely exceptionally effective for sucking prey from difficult-to-access cracks and crevices [32, 34, 35]. The presence of hypertrophied lips could also form the basis of mate choice and ultimately speciation [36]. However, there is substantial plasticity in the size of the lips depending on the substrates individuals use during feeding, so the hypertrophied lip phenotype might be readily gained and lost during evolution [37].

**Fig. 2:**
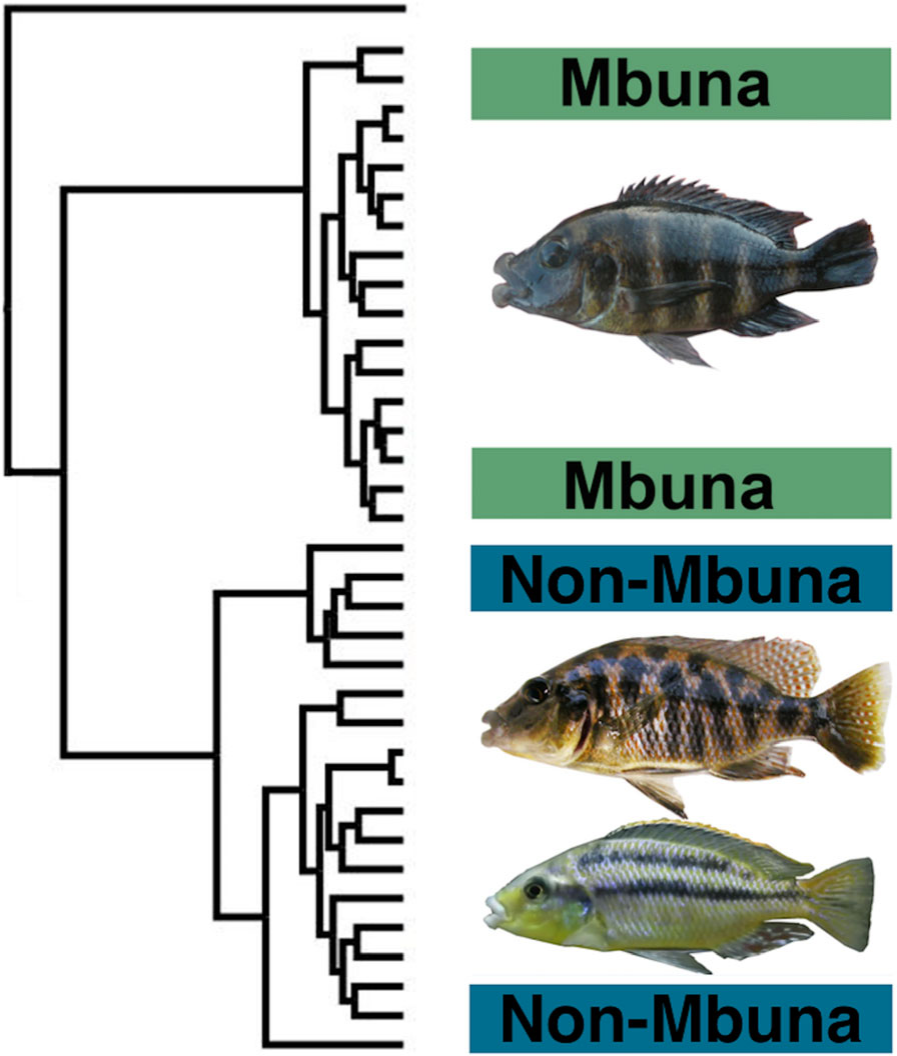
Phylogenies of Malawi cichlids based largely on mitochondrial DNA have suggested that hypertrophied lip lineages are paraphyletic. This cartoon phylogenetic reconstruction of Malawi cichlids integrates the relationships inferred from numerous studies of Malawi phylogenetics that have primarily focused on mitochondrial genes [22, 45–49]. The hypertrophied lip species *Placidochromis milomo* has been inferred to be nested within the rock-dwelling mbuna radiation with high bootstrap support while the similarly hypertrophied lip species *Placidochromis ornatus* and *Cheilochromis euchilus* have been inferred to be nested within the largely sand-dwelling non-mbuna component of the Malawi radiation

The repeated evolution of hypertrophied lips across both a diversity of teleosts as well as its presence within numerous phylogenetically disparate cichlid lineages suggests that the hypertrophied lip lineages in Lake Malawi could have evolved convergently. With approximately 1000 species of haplochromine cichlids, the opportunity for the repeated origin of adaptive traits within the Malawi radiation is extensive [38–40]. Also, Malawi species with hypertrophied lips are currently classified into several different genera based in part on their extensive differences in body pigment patterns [27, 41–44]. For instance, the species *Cheilochromis euchilus* exhibits black horizontal stripes while *Placidochromis milomo* has vertical barred pigmentation that could indicate affinities with evolutionarily disparate Malawi clades (Fig. 1). Additionally, the Malawi hypertrophied lip species *Placidochromis milomo* has been inferred, based on mitochondrial DNA, to be phylogenetically nested within the rock-dwelling “mbuna” clade (Fig. 2) while other species with hypertrophied lips have been inferred to lie within the largely sand-dwelling, or non-mbuna, group of Malawi cichlids [22, 45–49]. However, phylogenies reconstructed using mitochondrial sequence markers have known limitations for phylogeny reconstruction [17, 18] and body pigmentation could often be a poor predictor of evolutionary affinities [31, 50].

Next-generation sequence based phylogenomic approaches have the potential to greatly clarify evolutionary patterns. For instance, sequence capture of large numbers of highly conserved regions of organismal genomes shared among evolutionary distant taxa, or ultra-conserved elements (UCEs), has been efficiently used to generate massive genomic data sets capable of resolving relationships at deep timeframes [51–56]. Additionally, one of the most compelling characteristics of UCEs for phylogenetic reconstruction is that the flanking regions increase in variant sites as the distance from the UCE center increases, thereby potentially allowing for resolution of nodes at more recent evolutionary timescales [13, 57, 58]. Yet, even with the ability to generate these massive datasets, the best approach to analyzing this type of sequence data in rapid radiations like Lake Malawi is largely unclear [13, 59, 60]. In Lake Malawi cichlids, single loci of from 500 to 1000 base pairs that are often used for phylogenetic reconstruction could frequently have only a few variable single nucleotide polymorphisms (SNPs) [23]. This lack of variation could make evolutionary inferences problematic. Furthermore, interspecific gene flow could be common in this largely sympatric radiation [17, 47, 49, 61] providing opportunities for extensive recombination during this group’s divergence among even the few SNPs that do exist in a single sequence. Because of the vagaries of molecular evolution, model misspecification, as well as recombination, SNPs even in close proximity could often have unique evolutionary trajectories [62, 63]. Therefore, in Lake Malawi cichlids, analyzing SNPs independently and as individual data points might provide tractable estimates of phylogeny.

However, it has become clear that incomplete sorting of loci among lineages has to be considered when reconstructing bifurcating phylogenetic relationships [64–67]. Malawi cichlids could provide a radiation that is exceptionally prone to gene tree discordance. This is not just because of the high level of incomplete lineage sorting expected in a recent radiation, but also because of the extensive sympatry of Malawi lineages that provides the opportunity for hybridization and the apparent lack of extensive post-zygotic incompatibilities [17, 23, 61, 68]. Therefore, methods that reconstruct the species tree while incorporating the potential for incomplete lineage sorting could provide an enhanced understanding of the Malawi phylogeny in general and the relationships among the hypertrophied lip species in particular. Furthermore, if hybridization was exceptionally rampant among hypertrophied lip species, we might view their reconstructed relationships differently than if hybridization was more common between hypertrophied lip species and other members of the Malawi cichlid radiation.

We used both sequence data and single nucleotide polymorphisms (SNPs) generated from genotyping a large number of UCE loci to infer the relationships among several lineages within Lake Malawi. First, we examined whether there was greater evidence for extensive paraphyly or alternatively monophyly of several lineages of Lake Malawi’s hypertrophied lip species using concatenation methods that reconstruct a bifurcating topology. We also examined whether the UCE derived sequences and SNPs could provide insight into the relationships among the species with hypertrophied lips. Additionally, using sampling across geographically disparate sample sites in Lake Malawi for several congeneric species, we examined whether data from our UCE marker sets provided phylogenetic support for a number of taxonomically diagnosed clades using both concatenated analyses as well as species tree reconstructions that account for incomplete lineage sorting. Finally, we tested the support for hybridization among sampled members of the Malawi cichlid radiation and emphasized inferences of hybridization involving species with hypertrophied lips.

## Materials and methods

All fishes sequenced in this study were collected from Lake Malawi in 2010 using SCUBA and barrier nets. Using a combination of 23 newly sequenced individuals and published sequences for 25 individuals, phylogenetic relationships among 35 species of Malawi cichlid were examined (Table 1). Our samples include five of the seven currently known Malawi species with hypertrophied lips. Multiple individuals of *Placidochromis milomo*, *Cheilochromis euchilus*, and *Eclectochromis ornatus* were sampled*.* Because of availability, only one individual of the hypertophied lip taxa *Placidochromis* “Mbenji fatlip” and *Lichnochromis acuticeps* were analyzed. *Chilotilapia rhoadesii* that is not a species that exhibits hypertrophied lips but is thought to be closely related to *Cheilochromis euchilus* was also examined [58]. Additionally, we included two species of the genera *Labeotropheus, Pseudotropheus, Ctenophyarnx*, *Otopharynx,* and *Taeniolethrinops* as well as three members of the genera *Mylochromis*, *Placidochromis*, and *Nimbochromis*. The East African species *Pundamillia pundamillia*, *Haplochromis burtoni,* and *Simochromis babaulti* were used as outgroups to polarize relationships*.* We reconstructed phylogenies using UCEs that show substantial conservation across teleosts and represent loci that have been sequenced previously for African cichlid fishes [13, 58]. Therefore, they should not show particular bias in their amplification or sequence divergence in the Malawi cichlids sampled.

**Table 1:** Sampling of Specimens from Lake Malawi

Species	Number of Loci	Sampling Location	Genbank Accession
*Astatotilapia burtoni*	1047	commercial	[58]
*Aulonocara stuartgranti*	1042	Maleri	[58]
*Cheilochromis euchilus*	1042	Mbenji	[58]
*Cheilochromis euchilus*	1004	Thumbi West	KARF00000000
*Cheilochromis euchilus*	1007	Mumbo	KAUH00000000
*Chilotilapia rhoadesii*	1042	commercial	[58]
*Ctenopharynx nitidus*	1012	Choifu Point	KATA00000000
*Ctenopharynx pictus*	1026	Thumbi West	[13]
*Cyrtocara moorii*	1026	Thumbi West	KARL00000000
*Docimodus evelynae*	1024	Boadzulu	[13]
*Eclectochromis ornatus*	1024	Maleri	KATR00000000
*Eclectochromis ornatus*	1024	Maleri	KATS00000000
*Eclectochromis ornatus*	1002	Maleri	KAUJ00000000
*Eclectochromis ornatus*	1020	commercial	KAUI00000000
*Fossorochromis rostratus*	1040	Maleri	[58]
*Hemitilapia oxyrhyncha*	1018	Mumbo	[13]
*Labeotropheus fuelleborni*	1036	Thumbi West	[58]
*Labeotropheus trewavasae*	1036	Thumbi West	[58]
*Lichnochromis acuticeps*	1016	Thumbi West	KASA00000000
*Mchenga conophoros*	1024	Otter Point	[13]
*Melanochromis auratus*	1039	Thumbi West	[58]
*Mylochromis anaphyrmus*	1009	Harbor	KASU00000000
*Mylochromis epichorialis*	1008	Mbenji	KASV00000000
*Mylochromis mola*	1008	Otter Point	KASB00000000
*Nimbochromis linni*	1021	Thumbi West	KASW00000000
*Nimbochromis livingstonii*	1017	Chiofu Point	KASX00000000
*Nimbochromis polystigma*	1042	Otter Point	[58]
*Nyassochromis prostoma*	1003	Mazinizi Reef	KASY00000000
*Otopharynx heterodon*	1013	Harbor	[13]
*Otopharynx lithobates*	1042	Mumbo	[58]
*Placidochromis electra*	1037	Chiofu Point	[58]
*Placidochromis milomo*	1049	Chinyamwezi	[58]
*Placidochromis milomo*	1004	Thumbi West	KATM00000000
*Placidochromis milomo*	1013	Thumbi West	KATN00000000
*Placidochromis milomo*	1001	Maleri	KATO00000000
*Placidochromis milomo*	1019	Nakatenga	KATP00000000
*Pladichromis “*Mbenji fatlip*”*	1012	Mbenji	KATQ00000000
*Pseudotropheus crabro*	1046	Maleri	[58]
*Pseudotropheus flavus*	1042	Chinyankwezi	[58]
*Pundamilia pundamilia*	1047	commercial	[58]
*Rhamphochromis longiceps*	1047	commercial	[58]
*Simochromis babaulti*	1047	commercial	[58]
*Stigmatochromis woodi*	1025	Maleri	[13]
*Stigmatochromis woodi*	1018	Chiofu Point	KATV00000000
*Taeniolethrinops furcicauda*	1009	Chiofu Point	KATW00000000
*Taeniolethrinops praeorbitalis*	1011	Otter Point	[13]
*Taeniolethrinops praeorbitalis*	1024	Otter Point	KATY00000000
*Tyrannochromis nigriventer*	1022	Thumbi West	[13]

Species names, the number of loci sequenced, sampling location and Genbank Accession information are given for all individuals analyzed. All locations reported from this study are specific locations within Lake Malawi. Genbank accessions for a number of individuals are provided in McGee et al. [58] and Hulsey et al. (2017)

### DNA extraction and library preparation

We extracted DNA from 5 to 15 mg of ethanol-preserved tissues. We followed a modified version of the Qiagen DNEasy protocol, which utilizes 65 uL of warm (50–55 °C) buffer AE instead of the recommended 200 uL at room temperature. Following elution, we quantified extraction efficiency using a Qubit 2.0 Fluorometer by thoroughly mixing 2.0 uL eluate with 198 uL of fluorescent dye solution. To ensure high-quality extracts, we visualized 50-100 ng of each extract via electrophoresis using a 1.5% agarose gel in TBE. We then prepared 100 uL aliquots for each specimen that were equilibrated to a DNA concentration of 10 ng/uL and then sonicated the aliquots using a BioRuptor (Diagenode, Inc.). Each sample was sheared to generate products of 300–500 bp in length that were then size validated with gel visualizations.

Following sonication, we prepared libraries according to a modified version of the Illumina library preparation protocols from [52]. In preparing pooled DNA libraries, we used a series of standard library preparation reagents (Kapa Biosystems, Inc.) combined with dual-indexing adaptors [69] that we added during the PCR amplification phase. By doing so, we substantially reduced the number of primer tags needed to uniquely identify and differentiate libraries. Immediately after, we quantified the nucleic acid concentrations of pre-amplification libraries. Following quantification, we prepared a 50 uL PCR reaction mix consisting of 15–20 uL DNA library, 25 uL HiFi HotStart ReadyMix polymerase, 5 uL primer mix, and 0–5 uL double-distilled water (ddH2O). The following thermal cycle configuration was used: 98 **°**C for 45 s, 10–16 cycles of 98 **°**C for 15 s, 60 **°**C for 30s, 72 **°**C for 60s, then 72 **°**C for an extended 5 min, and an indefinite hold at 4 **°**C. As a final step, we purified resulting reactions with 1.8X Serapure solution [69], two 80% EtOH washes, and rehydrated purified samples with 23 uL10-mM Tris buffer.

### Library enrichment and sequencing

To prepare libraries for enrichment, the libraries were combined into pools of equimolar ratios (~ 500 ng per pool). To normalize the volumes of each pool, pools were dried down in a SpeedVac and rehydrated in 3.4 uL Tris buffer. Based on the sequence capture protocol available at ultraconserved.org, libraries were enriched for UCE targets using the following reagents: (1) 100 ng of the MYBaits UCE Capture Kit baits (MYcroarray, Inc.) (2) 500 ng blocking oligos designed against our custom dual sequence indexes, (3) MYcroarray MySelect hybridization solutions (MYcroarray, Inc.), and (4) 1% SDS (versus 10% SDS). The hybridization reaction was run for 24 h at 65 **°**C, allowing the capture probes to bind to UCE targets. Upon completion, we thoroughly mixed streptavidin-coated beads (MyOne C1, Life Technologies, Inc.) with the hybridized pools and then washed the bound libraries according to the protocol. Beads were then rehydrated in 33 uL of ddH2O, amplified with 15 uL of the mix in a post-hybridization limited cycle PCR recovery step, and the end products quantified using a Qubit fluorometer [52]. After qPCR-quantification of the enriched, double-indexed pools using a library quantification kit (Kapa Biosystems), we created an equimolar solution of all pools at a total concentration of 10 nM. These libraries were then shipped to the Georgia Genomics Facility and sequenced using the Illumina NextSeq PE150 platform.

### Sequence data assembly and alignment

Following sequencing, we trimmed adapters, low quality bases, and sequences containing ambiguous base calls using the Illumiprocessor tool [70] that provides a wrapper for the trimmomatic package [71]. The reads were assembled on a species-by-species basis into contigs using Trinity v2013-02-25 [72]. Following assembly, the PHYLUCE software package [73] containing custom Python code that integrates LASTZ to align species-specific contigs to the set of UCE probes was used for enrichment [53, 58]. This program creates a relational database of matches to UCE loci sorted by taxon. After generating the relational database of matches to enriched sequences and genome-enabled taxa, we used additional components of PHYLUCE to query the database and generate fasta files for the UCE loci we identified across all taxa [73]. Following enrichment and sequencing, contigs that either matched no UCEs or contigs that matched multiple loci were removed. Using the remaining set of contigs, a matrix was generated that included only UCE loci that were recovered from at least 95% of the species examined. The data are available on Genbank’s short read archive database (Table 1).

### Concatenated phylogeny reconstruction

To reconstruct phylogenetic hypotheses from our data, we concatenated our UCE alignments (Additional file 1) into a PHYLIP-formatted super-matrix [74].

We first carried out phylogenetic reconstruction on a 95% complete matrix with a GTR + gamma partitioning scheme using RAxML 8.0.19 [75] and the PTHREADS binary. Initially, 20 maximum-likelihood (ML) searches were conducted to find the best-fitting phylogenetic hypothesis. Then, we generated non-parametric bootstrap replicates under the autoMRE flag which runs the analysis until convergence. Upon completion, the best fit ML tree was reconciled with the bootstrap replicates to generate node support values.

To reconstruct phylogenetic trees using SNPs, we aligned all raw reads against the sample with the highest coverage across all UCE loci and utilized a de novo SNP calling approach as described in Hulsey et al. 2017 [13]. This method integrates BWA v. 0.7.7–1 and PICARD v. 1.106 (http://broadinstitute.github.io/picard//) to output alignments in BAM format, repairs any formatting violations, adds read group header information, and marks duplicates in each BAM. We then merged all resulting BAMs into one file, realigned the data, and called SNPs using GATK v. 3.5. To ensure high-quality SNPs in downstream analyses, the data was hierarchically filtered according to stringent quality and validation parameters, excluding SNPs with quality scores under 25, low variant confidence, and poor validation. Finally, the resulting data was filtered further using VCFTOOLS v. 0.1.14 [76] to remove all loci that missed SNP calls for over 25% of the species (Additional file 2).

We reconstructed SNP trees using two data sets. The first data set included all SNPs recovered with a minor allele frequency greater than 2%. Because linkage disequilibrium of SNPs in the same locus could influence our phylogenetic results, the second data set was filtered to only include the highest quality SNP per locus, resulting in 1024 SNPs (Additional file 3). We then converted the SNP data format from genomic data structure to FASTA via R packages “gdsfmt” and “SNPRelate” [77]. Then, we created a Phylip interleaved alignment file (Additional file 4) and ran the file through the PHYLIP program DNAML to infer a maximum likelihood tree [77]. Subsequently, 1000 non-parametric bootstrap replicates of the maximum likelihood tree were generated using the *bootstrap.pml* function in the R package “phangorn” as implemented in the SNPhylo pipeline [78, 79]. As a final step, the maximum likelihood phylogeny was reconciled with the bootstrap replicates to compute node support values.

### Species tree reconstruction

Using our conservative data set of a single SNP per locus, we reconstructed a species tree that accounts for incomplete lineage sorting using the coalescent-based SVDquartets program [80], implemented in PAUP v4.163 [81]. We evaluated all possible quartet combinations to produce the species tree. All individuals sampled from a species were used in the coalescent model allowing these individuals to inform the tree reconstruction. To assess confidence in nodes recovered, we generated 100 bootstrap replicate quartet trees from the 1024 SNPs.

### Testing for hybridization

We tested for the level of interspecific gene flow present in our sampling of Malawi cichlids and concentrated on inferences of gene flow involving the hypertrophied lip species. To do this, we used the program HyDe [82] to test for hybridization among all possible triplet combinations of species. HyDe uses phylogenetic invariants, similar to the D-statistic [83], to assess statistically significant evidence for hybridization. This was again implemented on our single SNP per locus data set of 1024 SNPs. Specifically, we employed the python script run_hyde.py to test all possible triplet comparisons among our sampled cichlids. Because all the possible triplet combinations for 37 taxa $\left(\genfrac{}{}{0pt}{}{37}{3}\right)\ x\ 3=$23,310 hypotheses tests, we assessed significance using a Bonferroni correction of (0.05)/(23,310) = 2.15 × 10^− 6^.

## Results

Following enrichment and sequencing, an average of 4,910,117 reads and 94,430 contigs were obtained per species. The analyzed matrix included only loci that were recovered from at least 95% of the species examined, constituting 1024 UCEs that had an average length of 324 bp. The assembled alignments included 563,696 base pairs of sequence. The alignments contained 10,465 total SNPs prior to filtering, 2707 when only SNPs with a minor allele frequency greater than 2% were included, and 1024 when only one SNP per locus for the 1024 UCE loci were included. We then generated phylogenies using RAxML on sequences (Additional file 5) as well as SNPhylo for the 2707 SNPs (Additional file 6), and for the 1024 SNPs (Additional file 7).

**Fig. 3:**
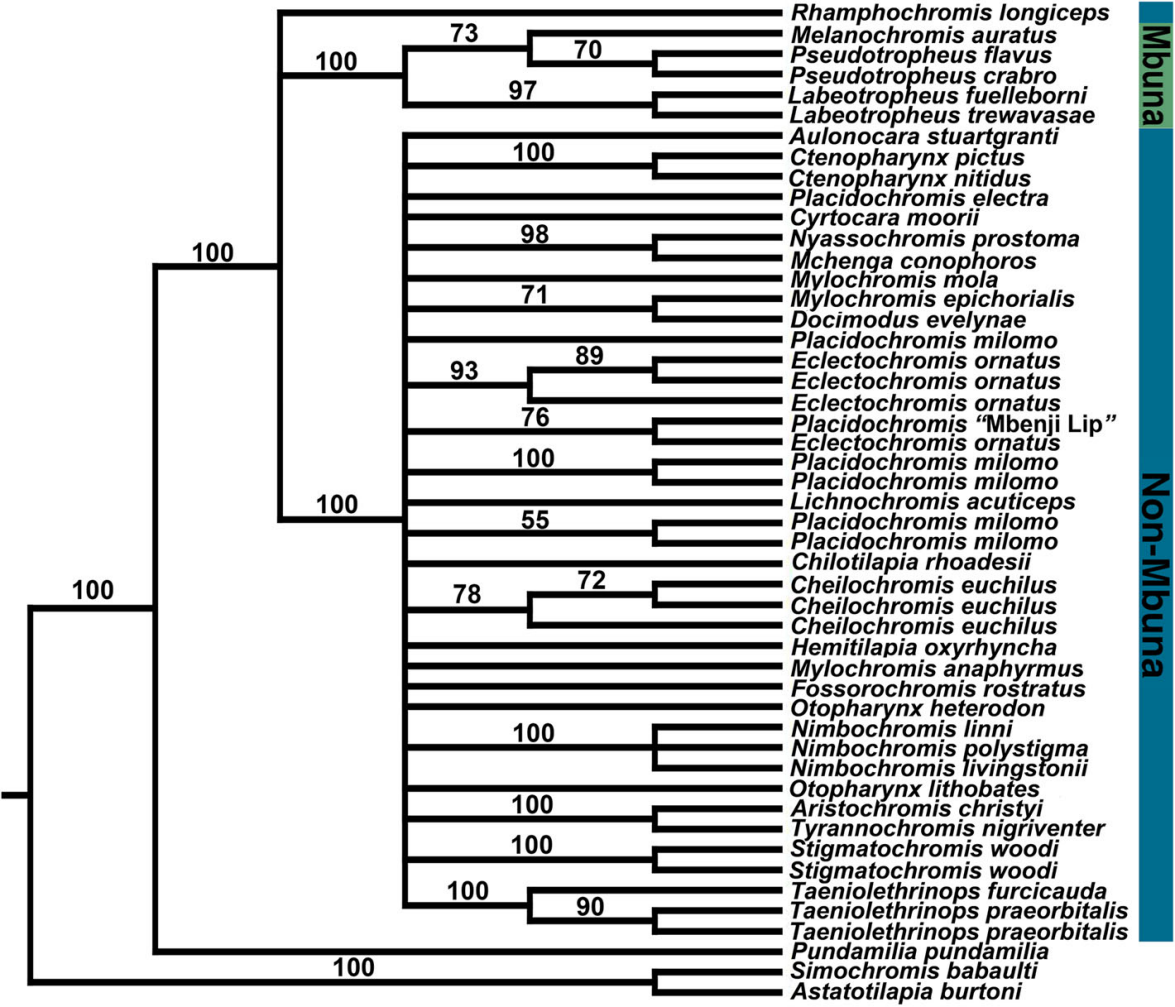
RAxML phylogenetic reconstruction of Malawi cichlds using 1024 UCEs. The reconstruction was based on a matrix was that included only UCE loci that were recovered from at least 95% of the species examined. The consensus topology is shown and bootstrap values greater than 50% from the combined searches are given behind the nodes. In general, there was clear support (100%) for the monophyly of both an mbuna clade and a large non-mbuna clade that included all of the species with hypertrophied lips. Many of the relationships among the non-mbuna, including those for the hypertrophied lip species, were not resolved here although species with multiple individuals sampled were often recovered as monophyletic

The RAxML inferred phylogeny for all the loci was generally less resolved than the SNP inferred phylogenies (Fig. 3). However, there was clear bootstrap support (100%) for both a monophyletic mbuna clade and a large non-mbuna clade that included all of the species with hypertrophied lips in the RAxML tree. Also, species with multiple individuals sampled were often recovered as monophyletic. A few notably supported nodes (100%) included those for the monophyly of the species sampled from the genera *Nimbochromis*, *Ctenochromis*, *Taeniolethrinops*, and *Labeotropheus*. Yet, many of the relationships among the non-mbuna, including those for the hypertrophied lip species, were not well-resolved (< 50% bootstraps) in this RAxML inferred phylogeny. Also, only the relatively poorly supported (71%) relationship of *Docimodus evelynae* and *Mylochromis epichoralis* was not recovered in the SNP inferred phylogenies.

**Fig. 4:**
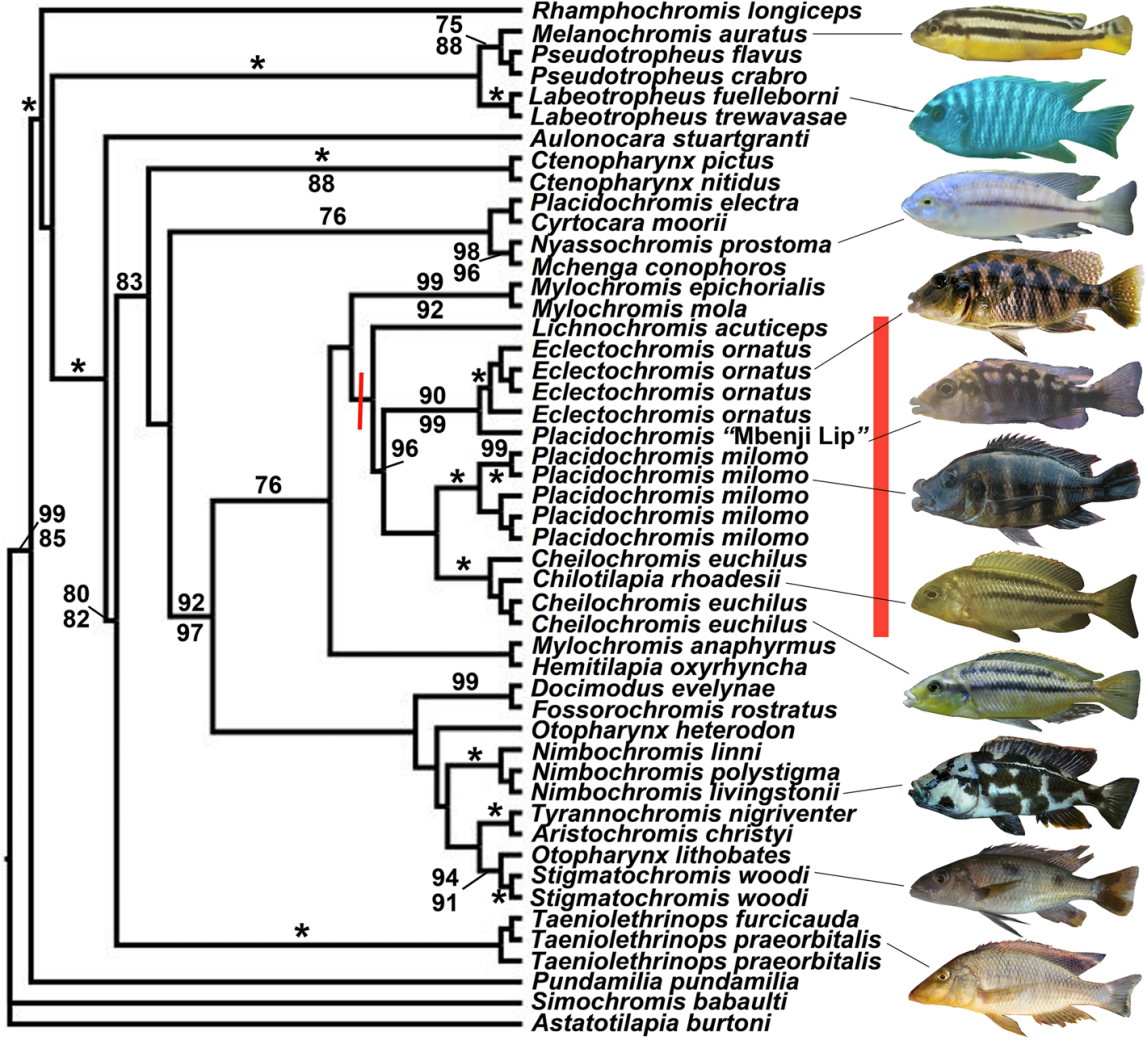
Phylogenetic reconstruction of UCE generated SNPs. The consensus topology inferred using the 2707 SNPs that had a minor allele frequency greater than 2% is presented. The bootstrap values of the 2707 SNP dataset are shown above the branches subtending nodes, and bootstraps from the data set limited to a single SNP per locus are shown below the branches. There is evidence for the monophyly, shown with a bar dissecting the branch that subtends their last common ancestor, of the hypertrophied lip species *Eclectochromis ornatus, Placidochromis “*Mbenji fatlip*”, Placidochromis milomo*, *and Cheilochromis euchilus* as well as the non-hypertrophied lip species *Chilotilapia rhoadesii*. This group is highlighted with a red bar behind the species names. Also, *Lichnochromis acuticeps*, which has somewhat hypertrophied lips, groups with these taxa greater than 50% of the time in the 2707 SNP dataset

Our concatenated SNP tree reconstructions provided novel phylogenetic inferences of Malawi cichlid evolutionary relationships (Fig. 4). We recovered strong support for *Rhamphochromis longiceps* as sister to the remaining diversity of the Malawi cichlids examined. As in the RAxML tree and a previous study [13], we recovered the relatively unambiguous monophyly of two major clades that respectively contained the rock-dwelling mbuna sampled and a clade of primarily sand-dwelling non-mbuna cichlids. The cichlids with hypertrophied lips were all recovered as nested within this non-mbuna clade. In general, the data sets analyzed using both the 1024 loci with RAxML and 1024 SNPs were generally consistent but provided less support than the relationships inferred using the 2707 SNP data set which we report on in detail below.

The 2707 concatenated SNPs supported the monophyly of a clade containing the species *Placidochromis milomo, Placidochromis* “Mbenji fatlip”*, Eclectochromis ornatus,* and *Cheilochromis euchilus* as well as the non-hypertrophied lip species *Chilotilapia rhoadesii*. *Lichnochromis acuticeps* grouped with these taxa greater than 50% of the time in the data set of 2707 SNPs. Within the hypertrophied lip group of species, *Placidochromis* “Mbenji fatlip” was recovered as sister to the monophyletic sampling of *Eclectochromis ornatus* (90% bootstraps in the larger SNP dataset). The individuals of *Placidochromis milomo* were also recovered as monphyletic (100%). In greater than 50% of the trees reconstructed using the 2707 SNPs, *Placidochromis milomo* was sister to the grouping of *Cheilochromis euchilus* + *Chilotilapia rhoadessi*.

In addition to the hypertophied lip clade, a number of taxonomically diagnosed lineages were also recovered as monophyletic with close to 100% bootstrap support. The members of the genus *Labeotropheus* were found to be monophyletic (100%). Additionally, both species of *Taeniolethrinops* were recovered as monophyletic (100%) as were the two individuals of *Stigmatochromis woodi* (100%) sequenced. Furthermore, the three species of *Nimbochromis*, *N. linni*, *N. polystigma*, and *N. livingstonii* formed a strongly supported monophyletic clade (100%).

A number of other relationships recovered were noteworthy. *Aulonocara stuartgrantii* was recovered as the sister group to the remainder of the large clade of non-mbuna. *Placidochromis* was not a monophyletic genus, as *P. electra* had strong affinities to *Cyrtocara moorii* and did not group with the hypertrophied lip *Placidochromis* species. *Mylochromis mola* and *M. epichorialis* were recovered as monophyletic, but *Mylochromis anaphyrmus* had somewhat unclear affinities with these other *Mylochromis*. As previously documented [13], *Tyrannochromis nigriventer* and *Aristochromis christyi* were found to form a clade (100%). Likewise, *Docimodus evelynae* and *Fossorochromis rostratus* were strongly supported (99%) as closely related. The SNPs provided substantial resolution not only for taxonomically recognized groups but also for some clades that have not been proposed previously.

**Fig. 5:**
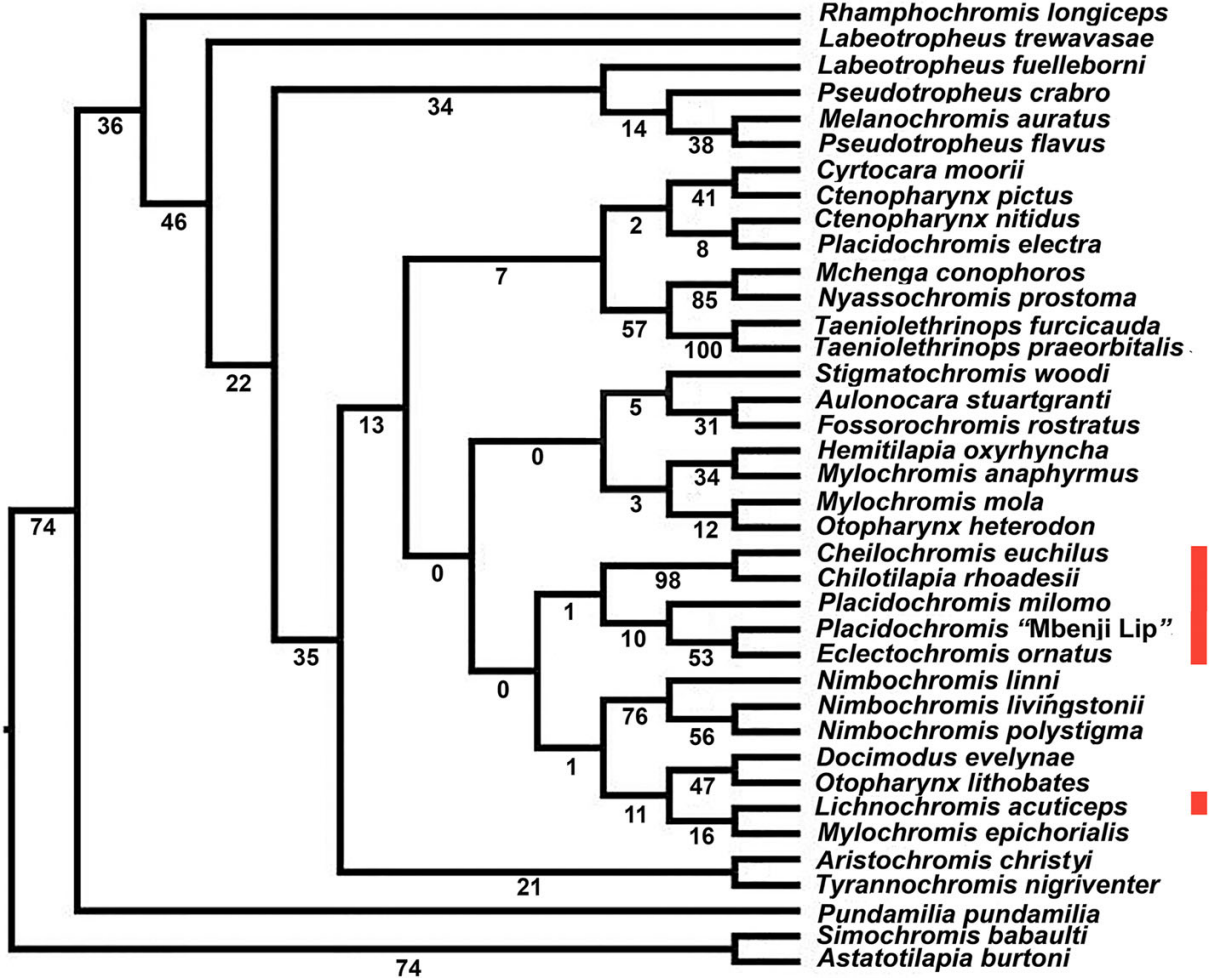
Quartet inferred species tree. To account for incomplete lineage sorting, we reconstructed the species tree of the Malawi cichlids sampled using the one SNP per locus data matrix analyzed in SVDquartets. The phylogeny inferred using the complete data matrix is shown, and bootstrap replicates of the data were used to generate the support values shown behind each node recovered in the original data matrix tree. All individuals sampled from a species were used in the coalescent model. This allowed these individuals to inform the species tree reconstruction, but in the above diagram they are collapsed into their respective species. Few of the nodes exhibit substantial bootstrap support. The species exhibiting hypertrophied lips are again highlighted with a red bar to the right of the species names

The SNP species tree reconstruction using SVDQuartets provided reduced resolution of relationships among lineages (Fig. 5). Following bootstrapping of SNPs, a limited number of clades were recovered in greater than 50% of the replicates. A sister group relationship of *Nimbochromis livingstonii* and *N. polystigma* were supported (56%) and their inclusion in a clade with *N. linni* was better supported (76%). *Mchenga conophoros* and *Nyassochromis prostoma* were recovered as having fairly strong phylogenetic affiinites (85%) and were minorly supported (57%) as aligned to a monophyletic group containing *Taeniolethrinops furcicauda* and *T. praeorbitalis. Pladichromis “*Mbenji Lip*”* and *Eclectochromis ornatus* was supported as a clade in 53% of reconstructions while *Cheilochromis euchilus* and *Chilotilapia rhoadesii* were recovered as sister in 98% of reconstructions. However, *Lichnochromis acuticeps* was not recovered as part of a hypertrophied lip clade. Nevertheless, the remaining hypertrophied lip clade was recovered as monophyletic, but in general there was limited resampling support (< 50%) for the existence of a monophyletic hypertrophied lip species tree clade.

Following the Bonferroni correction for multiple comparisons (23,310 comparisons) among all possible triplets (Additional file 8), none of the tests for introgression remained significant. Nevertheless, since introgression in the Malawi cichlids seems to be a potential factor in inferring evolutionary histories, we examined the top 50 most significant cases for introgression further. When we examined these 50 triplets, 12 of the top 50 inferences involved hypertrophied lip species (Table 2). However, only two of these involved triplets containing more than one hypertrophied lip species. Interestingly, in both cases the marginally hypertrophied lip species, *Lichnochromis acuticeps,* was inferred to be involved. However, in general there was no overwhelming evidence that the other hypertrophied lip species were exhibiting substantial introgression with one another.

**Table 2:** HyDe inferences of Malawi cichlid hybridization

P1	Hybrid	P2	*P*-value
*P. flavus*	*O. heterodon*	***P.*** **“Mbenji fatlip”**	0.0015
*L. trewavasae*	*H. oxyrhyncha*	***E. ornatus***	0.0022
*L. trewavasae*	*H. oxyrhyncha*	***P.*** **“Mbenji fatlip”**	0.0023
*L. trewavasae*	*M. mola*	***E. ornatus***	0.0040
*P. crabro*	***L. acuticeps***	***P.*** **“Mbenji fatlip”**	0.0114
*A. stuartgranti*	*M. conophoros*	***P.*** **“Mbenji fatlip”**	0.0122
*T. praeorbitalis*	*M. conophoros*	***P.*** **“Mbenji fatlip”**	0.0124
***P.*** **“Mbenji fatlip”**	***E. ornatus***	***L. acuticeps***	0.0125
*L. trewavasae*	*P. electra*	***P.*** **“Mbenji fatlip”**	0.0144
*C. pictus*	*C. nitidus*	***P.*** **“Mbenji fatlip”**	0.0148
*P. electra*	*H. oxyrhyncha*	***C. rhoadesii***	0.0159
*R. longiceps*	*M. anaphyrmus*	***E. ornatus***	0.0174

Inferences of hybridization [82] among Malawi cichlids and members of the ‘hypertrophied lip’ clade as shown in bold. We examined introgression occurring between triplets of three species: 1) An initial donor species (P1), 2) a hybrid species containing putatively introgressed loci, and 3) a second hybridizing donor ‘hypertrophied lip’ species (P2). Out of the 23,310 triplicates analyzed involving all the Malawi species sampled (Additional file 8), we depict only those inferred relationships involving a hypertrophied lip clade species that was recovered in the top 50 most likely hybridization events based on the *P*-values obtained from HyDe

## Discussion

The lineages in Lake Malawi that have hypertrophied lips based on concatenated sequence analyses appear to all fall within a relatively closely related and largely monophyletic group (Figs. 3, 4). Contrary to the results from numerous studies with mitochondrial DNA [22, 45–49], all the species exhibiting hypertrophied lips that we sequenced are well-nested within the primarily sand-dwelling non-mbuna and none are nested within the mbuna. Our phylogenetic reconstructions employing more than 1000 loci from throughout the nuclear genome argue that there is only support for a single origin of the hypertrophied lip phenotype within the several hundred species that comprise the Lake Malawi cichlid radiation. Our results also lend credence to the idea that pigmentation is likely to be a questionable indicator of taxonomic affinities and phylogenetic relationships within Malawi cichlids [83]. Transformations from lateral to horizontal stripes characterize many lineages within East African cichlids [31, 50], and Malawi cichlids with hypertrophied lips could provide a model group to investigate the mechanisms involved in these changes in body patterning.

There remain several caveats to the apparent monophyly of the hypertrophied lip cichlid clade within Lake Malawi that we recovered. There are additional lineages of hypertrophied lips in Malawi that we did not sample [41, 44], and there could be additional lineages of cichlids with this lip phenotype remaining to be discovered in this radiation of up to 1000 species. For instance, a neighbor-joining reconstruction of whole genome sequences recently provided the inference that the species *Placidochromis johnstoni* and *Hemitaeniochromis spilopterus*, neither of which were sampled in this study and neither of which have hypertrophied lips, could be nested within the clade of hypertrophied lip Malawi cichlids [68]. Additionally, although *Chilotilapia rhoadesii* has long been thought to be allied taxonomically and phylogenetically with *Cheilochromis euchilus*, this non-hypertrophied lip species appears to be nested within the otherwise hypertrophied lip clade (Fig. 4). This suggests that the hypertrophied lip phenotype has likely been lost in this one species, but it could potentially indicate the repeated evolution of this phenotype in two closely related Malawi lineages. Furthermore, our phylogenetic reconstructions do enforce a strictly bifurcating topology and there is extensive retention of ancestral polymorphism and has likely been substantial introgression in the Malawi radiation [17, 47, 49, 61]. Therefore, a bifurcating topology could provide misleading evidence of particular relationships [13]. However, despite these reservations, our analyses do provide evidence that the hypertrophied lip Malawi lineages are all apparently confined to the non-mbuna and show a closer affinity than has been previously appreciated (Fig. 2).

The SNPs generated from the sequencing of the ~ 1000 UCE loci also provided substantial power when concatenated to support the monophyly of a number of previously diagnosed taxa (Fig. 4). Not only were congeneric species from several clades such as *Otopharynx*, *Nimbochromis*, and *Taeniolethrinops* recovered as monophyletic, but this dataset provided the power to phylogenetically group multiple individuals sampled from the same species (Figs. 3,4). This level of resolution might seem trivial, but mitochondrial markers consistently support paraphyletic relationships of Malawi species [17, 61] and the RAxML tree provided poor resolution among most groups (Fig. 3). Interspecific gene flow could also commonly blur the genetic distinctiveness of species [47, 49]. However, these reconstructions argue that despite the substantial sequence polymorphism shared among different lineages of Malawi cichlids [17, 18, 20, 22, 23], some putatively bifurcating evolutionary relationships could be recoverable with sufficient information from the nuclear genome.

Although a few relationships were robustly recovered (> 85% bootstrap support) in our species tree reconstructions (Fig. 5), our analyses incorporating incomplete lineage sorting provide little resolution of Malawi cichlid relationships. For instance, although the non-bootstrapped topology recovered a monophyletic hypertrophied lip clade minus *Lichnochromis acuticeps*, we only recovered this clade in a single bootstrap replicate. Additionally, based on our HyDe analyses (Table 2), there could be ample introgression among Malawi lineages, but none of them were significant after adjustment for the over twenty thousand comparisons made in the small subset of species sampled. Evaluating hybridization using phylogenies will only become more difficult if these hypotheses are evaluated when including additional species from this very species rich radiation. Furthermore, the lack of substantial evidence for introgression involving more than one member of the hypertrophied lip clade suggests hybridization is not an overt cause of the apparent close evolutionary affinities of these species. This all highlights that introgression in Malawi might be common [61, 68], but larger data sets that employ more sophisticated methods as well as clear a priori hypotheses of introgression will likely be necessary to reconstruct evolutionary relationships among the hundreds of Malawi cichlid species.

Because it is the most species-rich radiation of fishes in the world, the Lake Malawi cichlid radiation will continue to serve as a model of comparative phenotypic evolution [38]. However, many comparative analyses of these fishes have either discounted the importance of phylogeny when examining trait divergence or relied on the limited inferences of relationships available from mitochondrial gene trees [46, 48, 84–86]. With the advent of high throughput genotyping of markers such as UCEs, comparative analyses should be able to effectively leverage relatively robust phylogenetic hypotheses to make inferences concerning the number of times that particular traits have evolved within groups like the Lake Malawi cichlids [1–6]. For instance, our results using a large dataset of UCE loci support the hypothesis that hypertrophied lips might have only arisen once among the approximately 1000 species of Lake Malawi cichlids. Next generation sequence data will continue to shed new light on whether novel traits have evolved repeatedly or only a single time even in the most rapidly diversifying of adaptive radiations.

## Conclusions

The Lake Malawi cichlid radiation provides an unparalleled model of comparative phenotypic evolution. Contrary to mitochondrial DNA phylogenies, bifurcating trees based on our 1024 UCE loci supported close affinities of Lake Malawi lineages with hypertrophied lips. Yet, future analyses will have to both collect more data and use more sophisticated analyses to account for incomplete lineage sorting. Phylogenomic analyses will continue to provide powerful inferences about whether phenotypic novelties arose once or multiple times during adaptive radiation.

## Additional files

Additional file 1: PHYLIP-formatted super-matrix of UCE alignments. (PHYLIP 88800 kb)

Additional file 2: VCF file for 10,465 SNPs. (VCF 12737 kb)

Additional file 3: VCF file for 1024 SNPs. (VCF 1384 kb)

Additional file 4: Phylip interleaved alignment file of SNPs. (NEXUS 49 kb)

Additional file 5: RaXML phylogeny. (NEX 6 kb)

Additional file 6: 2707 SNP SNPhylo phylogeny. (NEX 1 kb)

Additional file 7: 1024 SNP SNPhylo phylogeny. (NEX 277 kb)

Additional file 8: HyDe output for all comparisons. (XLSX 3454 kb)

## Abbreviations

BAM: Binary format for storing sequence data
DNA: Deoxyribonucleic acid
PCR: Polymerase chain reaction
SCUBA: Self-contained underwater breathing apparatus
SNP: Single nucleotide polymorphism
TBE: Tris-Borat-EDTA
UCE: Ultraconserved elements
